# Swallowing kinematic analysis might be helpful in predicting aspiration and pyriform sinus stasis

**DOI:** 10.1038/s41598-022-05441-2

**Published:** 2022-01-25

**Authors:** Kuo-Chang Wei, Sheng-Hao Cheng, Ming-Yen Hsiao, Yu-Chen Wang, Chi-Hung Weng, Jo-Yu Chen, Tyng-Guey Wang

**Affiliations:** 1 Department of Physical Medicine and Rehabilitation, College of Medicine, National Taiwan University, Taipei, Taiwan; 2 Department of Physical Medicine and Rehabilitation, National Taiwan University Hospital, Taipei, Taiwan; 3 Department of Medical Imaging, National Taiwan University Hospital, Taipei, Taiwan; 4 AetherAI Co., Ltd., Taipei, Taiwan

**Keywords:** Physiology, Diseases, Medical research

## Abstract

Aspiration due to dysphagia can lead to aspiration, which negatively impacts a patient’s overall prognosis. Clinically, videofluoroscopic swallow study (VFSS) is considered the gold-standard instrument to determine physiological impairments of swallowing. According to previously published literature, kinematic analyses of VFSS might provide further information regarding aspiration detection. In this study, 449 files of VFSS studies from 232 patients were divided into three groups: normal, aspiration, and pyriform sinus stasis. Kinematic analyses and between-group comparison were conducted. Significant between-group differences were noted among parameters of anterior hyoid displacement, maximal hyoid displacement, and average velocity of hyoid movement. No significant difference was detected in superior hyoid displacement. Furthermore, receiver-operating characteristic (ROC) analyses of anterior hyoid displacement, velocity of anterior hyoid displacement, and average velocity of maximal hyoid displacement showed acceptable predictability for detecting aspiration. Using 33.0 mm/s as a cutoff value of average velocity of maximal hyoid displacement, the sensitivity of detecting the presence of aspiration was near 90%. The investigators therefore propose that the average velocity of maximal hyoid displacement may serve as a potential screening tool to detect aspiration.

## Introduction

Dysphagia is a critical clinical issue that can occur in various disease groups, including stroke, head and neck cancer, Parkinson’s disease, and motor neuron disease^1–4^. Aspiration resulting from compromised swallowing can lead to aspiration pneumonia, which increases mortality and negatively affects the quality of life of patients^5,6^.

The swallowing mechanism can be divided into the following four phases: the oral preparatory phase, oral propulsive phase, pharyngeal phase, and esophageal phase^7^. During the complex pharyngeal phase of swallowing, the anterior–superior displacement of the hyoid bone plays a significant role in securing the airway to prevent aspiration and the opening of the upper esophageal sphincter for the smooth forward passage of food^1,2^. Therefore, the evaluation of hyoid movement during swallowing is of great significance in clinical practice.

Clinically, the videofluoroscopic swallow study (VFSS) is considered the gold-standard instrument to determine physiological impairments of swallowing^3^. Currently published tools to categorize the findings of VFSS studies include the penetration–aspiration scale (PAS) and modified barium swallow impairment profile (MBSImP). The PAS is an eight-point interval scale that assesses airway invasion events during swallowing^4^. The MBSImP is a comprehensive tool that assesses 17 different physiological domains during swallowing, including the extent of pharyngeal residues^5^. Unfortunately, subjectivity of the scorer is a drawback of these tools.

A more objective analysis of swallowing biomechanical events is VFSS overlaid with swallowing kinematic evaluation. This is also known to have excellent interrater and intrarater reliability^6^. VFSS has several disadvantages, however, including radiation exposure, altered food consistency and sensory feedback due to the use of contrast medium, and limited patient accessibility^7^. Alternative objective analysis of swallowing biomechanical events, in particular hyoid bone displacement, includes ultrasound and accelerometry, which may have greater applicability in swallowing evaluation^8–10^. Results of previously published studies have implied a correlation between the extent of anterior or upward displacement of the hyoid bone, velocity and trajectory of hyoid movement during swallowing, and penetration, aspiration, and postswallow residues; however, no consensus has been achieved thus far^11–21^. Thus, it is worth exploring the relationship between hyoid bone kinematics and swallowing impairment to further expand the clinical implication of hyoid bone kinematics during swallowing.

In this retrospective study, we aimed to investigate the relationship between hyoid movements and aspiration, and pyriform sinus stasis using kinematic analysis of VFSS studies. We hypothesized that reduced kinematic parameters of hyoid displacement and velocity would be present in groups with aspiration or pyriform sinus stasis. We also propose that measures of hyoid bone displacement and velocity could be used to predict the presence of aspiration and pyriform sinus stasis.

## Materials and methods

### Data collection

In this study, we retrospectively reviewed the files of VFSSs collected from May 1, 2015, to May 1, 2020. The study protocol was conducted in accordance with the ethical principles of the Declaration of Helsinki and was approved by the National Taiwan University Hospital Research Ethics Committee (Institutional Review Board: 202006027RINC). The need of informed consent was waived by National Taiwan University Hospital Research Ethics Committee due to the retrospective nature of this study. The VFSSs were conducted in the lateral plane and recorded with 30 frames per second. All studies were performed at the Department of Medical Imaging of National Taiwan University Hospital. The thin barium sulfate was prepared with Baritop LV 300 g in 180 ml water. The thick and paste barium sulfate consisted of a 100 ml of Baritop LV suspension and 1.5 g and 2.5 g of thickener (neo-high toromeal iii), respectively. 5 ml of barium sulfate of 3 different consistencies was swallowed for one time respectively by each patient. We excluded files with difficult recognition of hyoid bone, an anteroinferior extreme point of the mandible bone, and C2 to C5 vertebral bodies.

### Image analysis

Personal information, including patient age, sex, and disease entity, was removed before we initiated the data analysis. One speech and language pathologist who was well trained in swallowing kinematic analysis sorted the video into three categories using the MBSImP and the PAS. The normal swallowing group was defined as those with a PAS score of 1 and MBSImP score of 0–1. Images with residue at the pyriform sinus were defined as having an MBSImP score of 2–4. Finally, the aspiration group was defined as those with a PAS score of 6–8.

**Figure 1:**
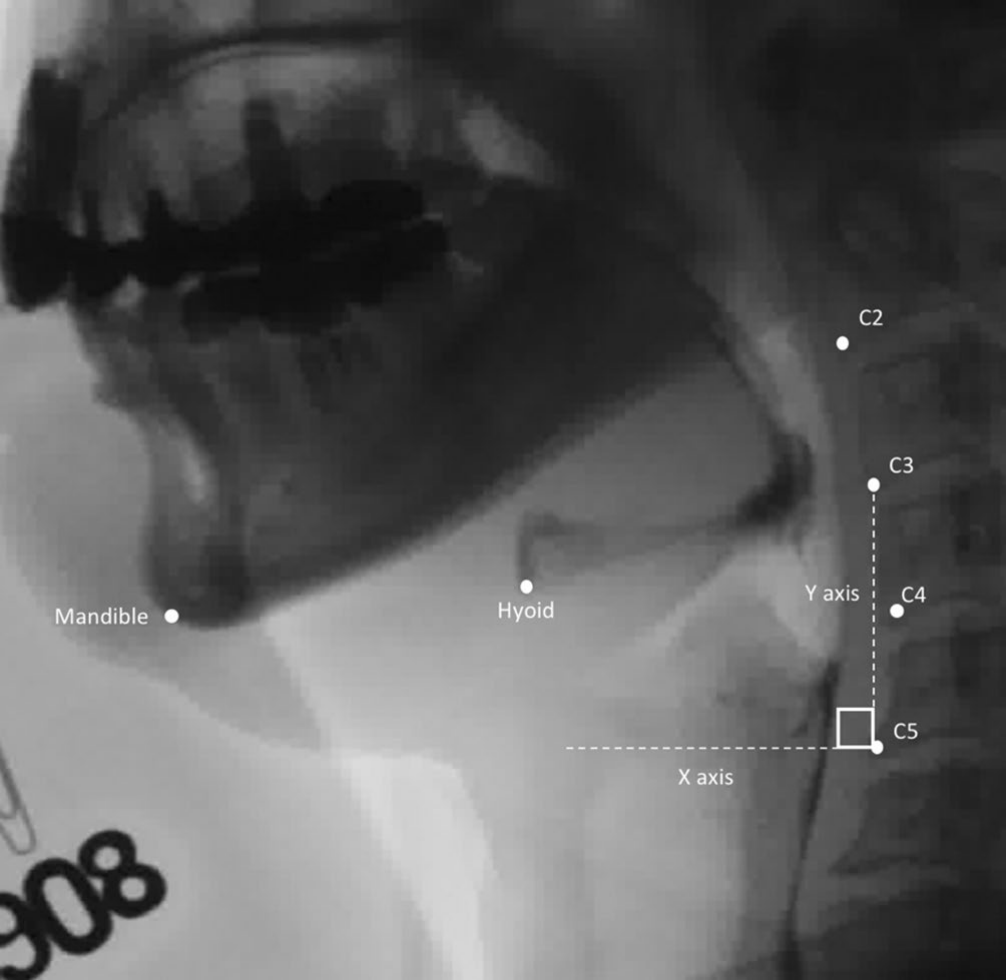
Points of interest and coordinate axis of VFSS image with Y-axis defined as the line connecting anterior–inferior corner of C3 and C5 vertebral bodies.

For labeling the points of interest on VFSS image, engineers of AetherAI Co., Ltd. constructed a web-based labeling interface software with the front-end framework built up with the Angular platform (Ver. 10.1.4) and the backend framework built up with Django framework (Ver. 2.2.10). With this web-based labeling software, users could not only mark the points of interest but also adjust the contrast of each image frame in order to improve the accuracy during image labeling. Two trained physiatrists (K.-C.W. and S.-H.C.) and two speech and language pathologists (Y.-C.W. and Y.-Y.P.) marked the following points of interest in each video frame using the AetherAI platform (Fig. 1): (1) anterior–inferior corner of the C2 vertebral body, (2) anterior–inferior corner of the C3 vertebral body, (3) anterior–inferior corner of the C4 vertebral body, (4) anterior–inferior corner of the C5 vertebral body, (5) anterior–inferior corner of the hyoid bone, and (6) the most prominent point at anterior–inferior corner of the mandible bone. To further conduct the hyoid kinematic analysis, a coordinate axis was defined with the anterior–inferior corner of the C4 vertebral body set as the origin. The *y*-axis was defined as the line connecting (2) and (4). The perpendicular line intersecting with (3) was defined as the *x*-axis. Based on the established coordinate axis, the anterior-inferior corner of the hyoid bone then possessed a position coordinate and parameters including the maximal displacement of hyoid bone during swallowing, the maximal displacement in the *x*-axis direction, and the maximal displacement in the *y*-axis direction were recorded. The maximal displacement of hyoid bone was the hypotenuse displacement from the origin calculated with Pythagorean theorem. The distance was measured by transforming the image pixels to corresponding millimeters (mm) and time duration between start and end points of hyoid movement in one swallow was recorded. The starting point was defined as recognizable initiation of hyoid motion while the end point was defined as termination of hyoid motion after swallowing. The average velocity of the hyoid bone movement including the x-vector and y-vector components were also calculated by dividing the hyoid bone displacement by the duration of hyoid movement.

### Statistical analysis

A preliminary Shapiro–Wilk test demonstrated that the data did not follow a normal distribution; thus, the Kruskal–Wallis test was used to compare the between-group differences of maximal displacement of the hyoid bone, maximal displacement of the hyoid bone in both the *x*- and *y*-axis directions, and the velocity of the hyoid bone displacement including the x-vector and y-vector components. All analyses were performed using SPSS version 25 (IBM, Armonk, NY). Significant between-group differences were defined as having a *P* value < 0.05. When the results of the Kruskal–Wallis test showed significant between-group differences, post hoc analysis using pairwise Mann–Whitney test was applied. To investigate whether the displacement and velocity of hyoid movement could be applied to further detect aspiration and pyriform sinus stasis, a receiver-operating characteristic (ROC) analysis was conducted as well. An area under curve (AUC) should at least surpass 0.7 to be considered to possess an acceptable discrimination ability^22^.

## Results

### Between-group difference

In total, 449 VFSS files from 232 patients were analyzed: 230 files in the normal group, 87 files in the aspiration group, and 132 files in the stasis group. The mean age of the enrolled patients was 64.7 years old. The disease entities were summarized in Table 1 as various kinds of patient groups including stroke, head and neck cancer, Parkinson’s disease and traumatic brain injuries were recruited in this study.

**Table 1:** Demographic data of VFSS files analyzed.

Disease entity	Number of videos (%)
Stroke	150 (33.4%)
Head and neck cancer	83 (18.5%)
Parkinson’s disease	34 (7.8%)
Traumatic brain injury	15 (3.3%)
More than one of the above	10 (2.2%)
Others	157 (34.8%)

Table 2 shows significant between-group differences among the parameters, including anterior hyoid displacement, maximal hyoid displacement, and average velocity of hyoid displacement. No significant difference was detected in superior hyoid displacement.

**Table 2:** Comparisons of swallowing kinematic parameters between the normal population and populations with aspiration and pyriform sinus stasis.

Parameters	Groups	N	Mean	First quartile	Third quartile	95% Confidence interval		p-value
						LL	UL	
Anterior hyoid displacement (mm)	Normal group	230	17.03	12.12	21.67	16.12	17.93	0.00
	Aspiration group	87	11.51	7.37	14.8	10.09	12.94	
	Stasis group	132	12.98	8.72	16.99	11.85	14.11	
Superior hyoid displacement (mm)	Normal group	230	20.63	13.3	26.96	19.19	22.07	0.426
	Aspiration group	87	19.73	12.22	24.85	17.24	22.23	
	Stasis group	132	18.88	12.84	24.13	17.35	20.4	
Maximal hyoid displacement (mm)	Normal group	230	29.85	21.26	33.09	27.47	32.25	0.00
	Aspiration group	87	25.37	18.31	28.64	21.09	29.65	
	Stasis group	132	23.69	18.15	28.33	22.14	25.24	
Velocity of anterior hyoid displacement (mm/s)	Normal group	230	19.18	7.78	25.47	17.18	21.19	0.00
	Aspiration group	87	7.88	2.05	9.51	5.88	9.88	
	Stasis group	132	15.76	7.8	20.02	13.9	17.62	
Velocity of superior hyoid displacement (mm/s)	Normal group	230	24.49	11.53	34.15	22.31	26.67	0.024
	Aspiration group	87	16.32	5.91	22.67	13.48	19.17	
	Stasis group	132	24.86	13.06	34.04	22.15	27.56	
Average velocity of maximal hyoid displacement (mm/s)	Normal group	230	28.27	14.73	40.41	27.01	29.53	0.00
	Aspiration group	87	16.48	5.75	23.3	13.23	19.73	
	Stasis group	132	29.54	16.99	38.46	26.12	32.95	

We conducted a pairwise comparison. Comparing the normal group and aspiration group, significantly greater anterior hyoid displacement, velocity of anterior hyoid displacement, velocity of superior hyoid displacement, and average velocity of maximal hyoid displacement were noted in the normal group. In the comparison between the normal group and stasis group, we found a significant between-group difference in anterior hyoid displacement and maximal hyoid displacement. For the comparison between the aspiration group and stasis group, we noted a significant between-group difference in the velocity of the anterior, superior, and maximal hyoid displacement.

### ROC analyses

**Figure 2:**
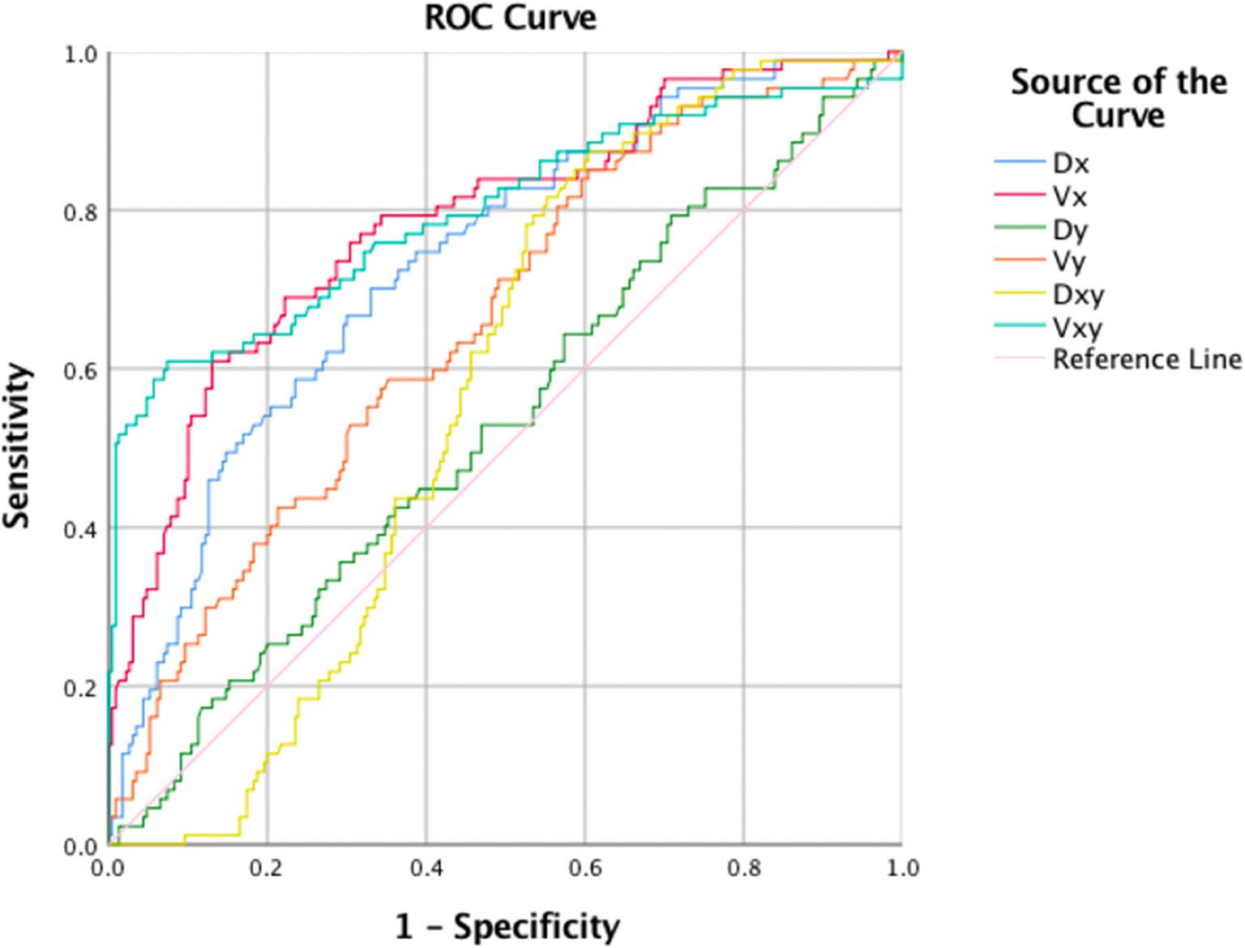
ROC curve of parameters of hyoid movement kinematic analyses for predicting aspiration. *Dx* anterior hyoid displacement, *Vx* velocity of anterior hyoid displacement, *Dy* superior hyoid displacement, *Vy* velocity of superior hyoid displacement, *Dxy* maximal hyoid displacement, *Vxy* average velocity of maximal hyoid displacement.

**Figure 3:**
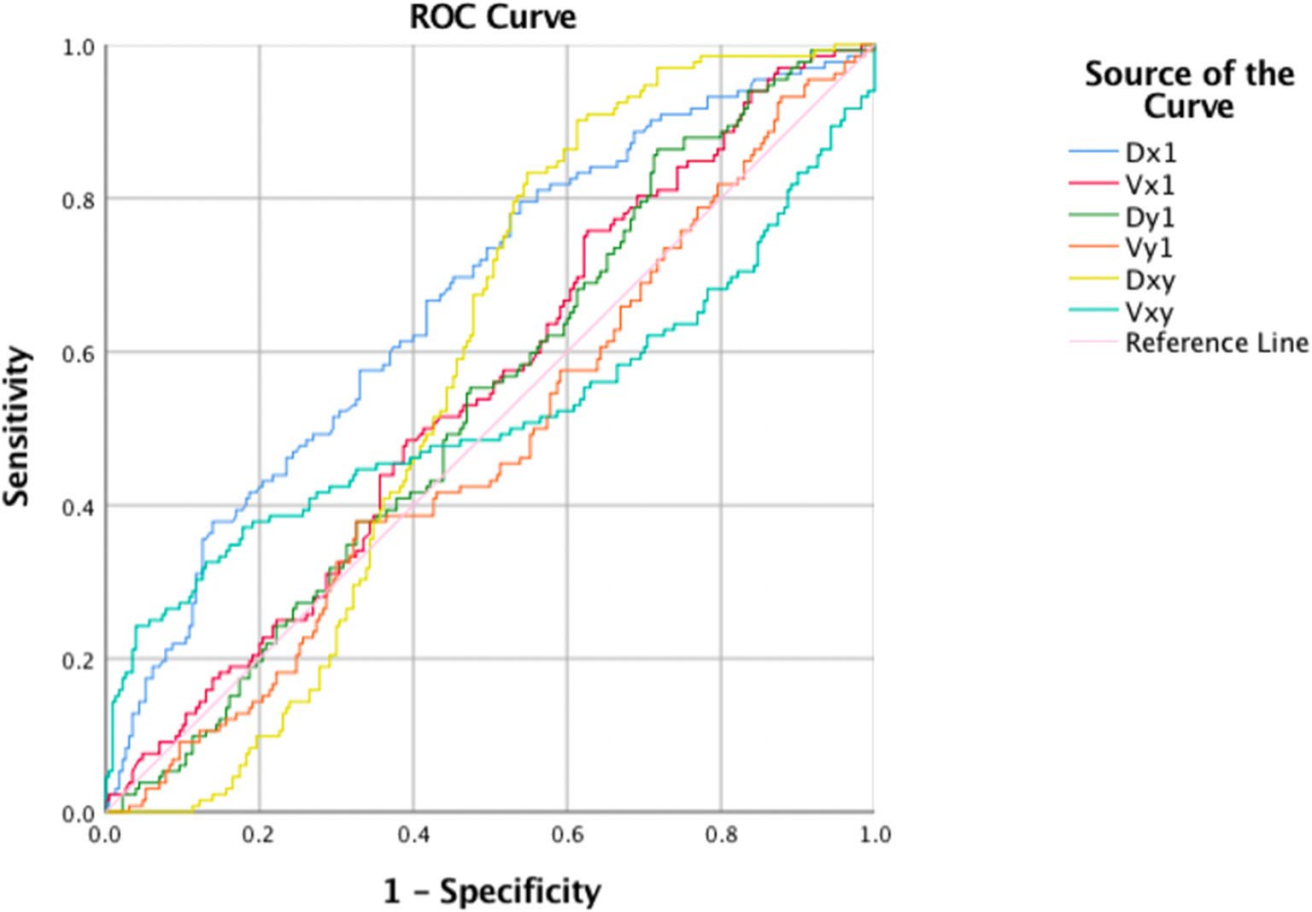
ROC curve of parameters of hyoid movement kinematic analyses for predicting pyriform sinus stasis. *Dx* anterior hyoid displacement, *Vx* velocity of anterior hyoid displacement, *Dy* superior hyoid displacement, *Vy* velocity of superior hyoid displacement, *Dxy* maximal hyoid displacement, *Vxy* average velocity of maximal hyoid displacement.

Figures 2 and 3 present the ROC curves assessing the predictability of hyoid kinematics for aspiration and pyriform sinus stasis, respectively. The AUC and best cutoff values are shown in Tables 3 and 4. The AUC of anterior hyoid displacement, velocity of anterior hyoid displacement, and average velocity of maximal hyoid displacement in Table 3 were 0.736 (cutoff value: 13.5 mm), 0.787 (cutoff value: 5.4 mm/s), and 0.798 (cutoff value: 33.0 mm/s) respectively, showing acceptable predictability for aspiration. For the prediction of pyriform sinus stasis, none of the obtained AUC values were greater than 0.7.

**Table 3:** AUC and corresponding cutoff value, sensitivity, and specificity obtained from the ROC analyses for predicting aspiration.

Measure	Cutoff value	AUC	Aspiration present	
			Sensitivity	Specificity
Anterior hyoid displacement (mm)	13.5	0.736	0.701	0.670
Superior hyoid displacement (mm)	26.3	0.532	0.793	0.287
Maximal hyoid displacement (mm)	10.8	0.572	0.17	0.99
Velocity of anterior hyoid displacement (mm/s)	5.4	0.787	0.609	0.87
Velocity of superior hyoid displacement (mm/s)	16.6	0.658	0.586	0.648
Average velocity of maximal hyoid displacement (mm/s)	33.0	0.798	0.874	0.396

**Table 4:** AUC and corresponding cutoff value, sensitivity, and specificity obtained from the ROC analyses for predicting pyriform sinus stasis.

Measure	Cutoff value	AUC	Stasis present	
			Sensitivity	Specificity
Anterior hyoid displacement (mm)	17.5	0.668	0.795	0.461
Superior hyoid displacement (mm)	24.5	0.538	0.864	0.283
Maximal hyoid displacement (mm)	33.5	0.576	0.902	0.387
Velocity of anterior hyoid displacement (mm/s)	20.1	0.550	0.758	0.374
Velocity of superior hyoid displacement (mm/s)	15.9	0.482	0.379	0.67
Average velocity of maximal hyoid displacement (mm/s)	15.8	0.520	0.24	0.86

## Discussion

The primary finding of this study was significant between-group differences for measurements of anterior hyoid displacement, maximal hyoid displacement, and velocity of hyoid movement among the normal, aspiration, and stasis groups. The ROC analyses and the corresponding AUC calculated from anterior hyoid displacement, velocity of anterior hyoid displacement, and average velocity of maximal hyoid displacement showed acceptable discrimination ability.

Abnormal swallowing kinematic parameters including anterior or superior hyoid displacement, timing of hyoid bone excursion, and velocity of hyoid bone movement during swallowing have been postulated as possible contributing factors for aspiration^11–20^. Several studies suggested that anterior hyoid displacement is related to pharyngeal processes during swallowing, including opening of the upper esophageal sphincter, whereas vertical displacement of the hyoid bone was highly variable because of the different resting positions in anatomical variations and its relation to oral processes only^23^. In this study, pairwise comparison between aspiration and normal groups showed a significantly lower value of anterior hyoid displacement and velocity of anterior hyoid displacement, superior hyoid displacement, and maximal hyoid displacement in the aspiration group. Nevertheless, whether there is a single convincing parameter of swallowing kinematics that leads to aspiration still requires further evidence.

Pyriform sinus stasis is a crucial factor that might be correlated with the severity of aspiration^24^. Reduced anterior movement of the hyoid bone can lead to insufficient opening of the cricopharyngeal muscle, causing stasis in the pyriform sinuses and aspiration after the swallow^24,25^. In the current study, significantly reduced anterior and maximal movement of the hyoid was measured when comparing the stasis group and the normal group. We further attempted to use kinematic parameters to correlate impaired hyoid movement with the presence of pyriform sinus stasis. Nonetheless, our data showed suboptimal correlation. One possible explanation for this is the impact of neuronal inhibition of the tonic cricopharyngeus muscle and intrabolus pressure needed to adequately open the cricopharyngeus muscle^25^. Previous studies have shown that VFSS can provide detailed information for structural and timely kinematic analyses of the cricopharyngeus muscle^26^. However, other tools including manometry or electromyography for the cricopharyngeal muscle might be needed to delineate the underlying process of inadequate cricopharyngeal muscle opening leading to pyriform sinus stasis^25^.

Only a few previously published studies have postulated that some parameters might be helpful in detecting aspiration^11,17,20^. Seo et al. analyzed multiple VFSS swallowing kinematic parameters among populations with poststroke dysphagia and found that the maximal tilt angle of the epiglottis had predictive value for the detection of aspiration^11^. According to the results of the study by Steele et al., the sensitivity of anterior hyoid displacement as a diagnostic parameter for detecting aspiration was as high as 90%^20^. In addition, maximum anterior hyoid displacement might predict the risk of penetration and aspiration according based on the research from Zhang et al., but the predicted and observed probability did not always match^17^. In the current study, acceptable predictability was attained while using anterior hyoid displacement and average velocity of maximal hyoid displacement to conduct ROC analyses, which was similar to the result from Steele et al.^20^. In addition, the results of our study showed that the sensitivity was close to 90% using 33.0 mm/s as the cutoff value for the average velocity of maximal hyoid displacement.

This study has several limitations. First, the VFSS protocol applied was not consistent with MBSImP protocol. The original MBSImP protocol used standardized, commercial preparations of barium contrast agents (Varibar^®^ E-Z-EM, Inc.) but this product was not available in our facility^5^. In order to erase the potential impact from different formula of barium sulfate used for VFSS, consistency of barium sulfate could be stratified using widely accepted terminology system such as International Dysphagia Diet Standardisation Initiative (IDDSI) in the future. Second, the analysis was not conducted in accordance with stratification of different food consistencies. However, conflicting results have been published from studies investigating in influence of bolus consistency on hyoid kinematics. Available evidence has shown that different bolus textures have a possible influence on hyoid movement, but the parameters of hyoid kinematics of normal adults remained stable across different bolus consistencies according to Steele et al. and Smaoui et al.^27–29^. Nonetheless, normal population was not enrolled in current study because those referred for VFSS exam in our facility were clinically suspected to have dysphagia. Therefore, the predictability of kinematic analysis of the hyoid bone in aspiration or pyriform sinus stasis with regard to specific food consistency still requires further research. Third, the groups analyzed in this research included a variety of disease populations. Our results therefore provide a generalized scope of using swallowing kinematic analysis to detect aspiration among a variety of disease populations. However, disease-specific analysis is needed to advance clinical practices for specific patient populations^11,30–32^. Fourth, the results of this study do not take into account other biomechanical events of swallowing including epiglottic movement, laryngeal movement, and pharyngeal constriction. Impairments in these swallowing events may also contribute to aspiration and pyriform sinus stasis^11,13,33^. Further studies are required to investigate other physiologic parameters and the potential to predict aspiration. Lastly, we did not conduct hyoid kinematic analysis for individuals with both aspiration and pyriform sinus stasis, which can occur simultaneously. To broaden the clinical implication of hyoid kinematic analysis for those at risk of dysphagia, further studies are needed for the populations suffering from various presentations of dysphagia.

### Ideas for clinical application and future research

The investigators of this study propose that assessing average velocity of maximal hyoid displacement using VFSS and/or submental ultrasound with kinematic analysis may be a useful screening tool for dysphagia evaluation. One drawback of VFSS exam is its limited accessibility^7^. Fortunately, hyoid kinematic analysis using submental ultrasound is a feasible alternative and able to be performed at bedside. In addition, measurements of submental US have been shown to correlate well with VFSS^34^. To expand the utility, further research is needed to investigate the diagnostic power of ultrasound examination compared with the results of the kinematic analysis obtained with VFSS studies.

## Conclusions

Significant between-group differences in anterior hyoid displacement and the velocity of hyoid displacement were observed in this study among the normal, aspiration, and pyriform sinus groups. ROC analysis revealed that anterior hyoid displacement and velocity of the anterior and maximal hyoid displacement had acceptable predictability in detecting aspiration. In addition, the sensitivity obtained when using 33.0 mm/s as the cutoff value for average velocity of maximal hyoid displacement was close to 90%. We assumed that the velocity of the maximal hyoid displacement could be used as a potential screening tool for the detection of aspiration. However, whether there is possible indicator of pyriform sinus stasis requires further evidence.

## Abbreviations

AUC: Area under curve
MBSImP: Modified barium swallow impairment profile
PAS: Penetration–aspiration scale
ROC: Receiver-operating characteristic
VFSS: Videofluoroscopic swallow study
